# Bioengineered Temporomandibular Joint Disk Implants: Study Protocol for a Two-Phase Exploratory Randomized Preclinical Pilot Trial in 18 Black Merino Sheep (TEMPOJIMS)

**DOI:** 10.2196/resprot.6779

**Published:** 2017-03-02

**Authors:** David Faustino Ângelo, Florencio Gil Monje, Raúl González-García, Christopher B Little, Lisete Mónico, Mário Pinho, Fábio Abade Santos, Belmira Carrapiço, Sandra Cavaco Gonçalves, Pedro Morouço, Nuno Alves, Carla Moura, Yadong Wang, Eric Jeffries, Jin Gao, Rita Sousa, Lia Lucas Neto, Daniel Caldeira, Francisco Salvado

**Affiliations:** ^1^ Centro Hospitalar de Setúbal, EPE Stomatology Department Setúbal Portugal; ^2^ Instituto Politécnico de Leiria Centre for Rapid and Sustainable Product Development (CDRSP) Leiria Portugal; ^3^ Faculdade de Medicina Universidade de Lisboa Lisboa Portugal; ^4^ University Hospital Infanta Cristina, Badajoz, Spain Department of Oral and Maxillofacial Surgery Badajoz Spain; ^5^ Kolling Institute Institute of Bone and Joint Research University of Sydney Sydney Australia; ^6^ Coimbra University Coimbra Portugal; ^7^ Faculdade de Medicina Veterinaria Interdisciplinary Centre of Research in Animal Health (CIISA) Lisbon University Lisboa Portugal; ^8^ Faculdade de Medicina Veterinaria Lisbon University Lisboa Portugal; ^9^ Instituto Nacional de Investigação Agrária e Veterinária (INIAV I.P.) Santarém Portugal; ^10^ University of Pittsburgh Chemical Engineering and Surgery Pittsburgh University of Pittsburgh Pittsburgh, PA United States

**Keywords:** temporomandibular joint disorders (TMD), temporomandibular joint bioengineered disk implants, temporomandibular randomized preclinical trial protocol

## Abstract

**Background:**

Preclinical trials are essential to test efficacious options to substitute the temporomandibular joint (TMJ) disk. The contemporary absence of an ideal treatment for patients with severe TMJ disorders can be related to difficulties concerning the appropriate study design to conduct preclinical trials in the TMJ field. These difficulties can be associated with the use of heterogeneous animal models, the use of the contralateral TMJ as control, the absence of rigorous randomized controlled preclinical trials with blinded outcomes assessors, and difficulties involving multidisciplinary teams.

**Objective:**

This study aims to develop a new, reproducible, and effective study design for preclinical research in the TMJ domain, obtaining rigorous data related to (1) identify the impact of bilateral discectomy in black Merino sheep, (2) identify the impact of bilateral discopexy in black Merino sheep, and (3) identify the impact of three different bioengineering TMJ discs in black Merino sheep.

**Methods:**

A two-phase exploratory randomized controlled preclinical trial with blinded outcomes is proposed. In the first phase, nine sheep are randomized into three different surgical bilateral procedures: bilateral discectomy, bilateral discopexy, and sham surgery. In the second phase, nine sheep are randomized to bilaterally test three different TMJ bioengineering disk implants. The primary outcome is the histological gradation of TMJ. Secondary outcomes are imaging changes, absolute masticatory time, ruminant time per cycle, ruminant kinetics, ruminant area, and sheep weight.

**Results:**

Previous preclinical studies in this field have used the contralateral unoperated side as a control, different animal models ranging from mice to a canine model, with nonrandomized, nonblinded and uncontrolled study designs and limited outcomes measures. The main goal of this exploratory preclinical protocol is to set a new standard for future preclinical trials in oromaxillofacial surgery, particularly in the TMJ field, by proposing a rigorous design in black Merino sheep. The authors also intend to test the feasibility of pilot outcomes. The authors expect to increase the quality of further studies in this field and to progress in future treatment options for patients undergoing surgery for TMJ disk replacement.

**Conclusions:**

The study has commenced, but it is too early to provide results or conclusions.

## Introduction

The temporomandibular joint (TMJ) is the most frequently used joint in the human body. The TMJ opens and closes 1500 to 2000 times daily and is essential for everyday functions of the mouth, such as mastication, speech, deglutition, yawning, and snoring, involving special mandatory synergy of both articular sides [1]. The TMJ disk is an essential component in the normal TMJ and has the following functions: (1) it distributes the intra-articular load, (2) it stabilizes the joints during translation, and (3) it decreases the wear of the articular surface [2,3]. The majority of TMJ disorders (TMD) are successfully treated with reversible, conservative, and low-tech treatments such as education and counseling, therapeutic exercises, splint therapy, and pharmacotherapy [4,5].

When the TMJ disk is displaced, malformed, or damaged, it can induce serious internal pathologic processes and/or osteoarthritis [6,7]. Currently, patients suffering from severe TMD have limited validated treatment options. Most surgical approaches, such as TMJ discectomy, do not restore the structural or biological properties of the articulation and disk. This procedure may not be ideal because the TMJ is left without an important functional structure. A variety of interpositional materials have been used to replace the removed disks, including synthetic materials manufactured from silicone, Teflon, polytetrafluoroethylene, and biological interpositional grafts taken from different anatomic sites [8-11]. These interpositional materials do not take in consideration the anatomy and biochemical and biomechanical characteristics of the TMJ native disk [12], and some of them have been associated with serious complications for the patients [8,13,14]. In the late 1980s, Proplast/Teflon TMJ (synthetic interpositional implant) were found to be harmful in many patients. The breakdown of the material, probably caused by TMJ high biomechanical forces, lead to fragmented particles that resulted in an immune foreign body response that caused problems ranging from severe cutaneous inflammatory reaction in the preauricular and cheek areas [15] to severe degenerative joint disease with perforation into the middle cranial fossa [16,17]. The result was a dramatic clinical spectrum of failures for these implants [10]. In December 1991, the US Food and Drug Administration’s Bulletin recommended immediate removal of all previous TMJ Proplast/Teflon implants because of the mechanical failures, many resulting in progressive bone degeneration [18]. In a 1992 workshop, the American Academy of Oral and Maxillofacial Surgery instructed the discontinuation of Proplast/Teflon [18].

The absence of efficacious options to substitute the TMJ disk can be related to difficulties in the translation of animal evidence to the clinical practice in humans. These limitations are likely related to:

1. the use of heterogeneous animal models with conflicting results, possibly due to variable anatomy and intra-articular loading between species [19,20];

2. the use of the contralateral TMJ as control, which may be associated with contralateral overloading [21];

3. the biomaterials used to replace the disk do not account for the morphologic and biomechanical characteristics of the native disk;

4. absence of randomized controlled trials with blinding of outcomes’ assessors; and

5. lack of multidisciplinary teams involved in the project.

Preclinical research should promote the effective translation of knowledge into practice. The previously mentioned aspects can limit the effective translation of quality scientific knowledge into clinical practice and these may present potential issues to patients, clinicians, and scientific progress.

The contemporary absence of successful options to substitute the TMJ disk is still a major issue for public health. Little has changed in the past decade regarding study designs for TMJ investigation, and the treatment for patients with severe TMD remains controversial. The main objective of the Temporomandibular Joint Interposal Material Study (TEMPOJIMS) is to develop a new, reproducible, and effective study design for preclinical research in the TMJ field. The second goal is to progress in bioengineering and regenerative medicine evaluating the benefits of a TMJ bioengineering implant to substitute the damaged native TMJ disk. This preclinical exploratory study is divided into two phases. Phase 1 of this study is a blinded randomized preclinical trial, designed to investigate if the TMJ undergoes important injury in bilateral discectomy, bilateral discopexy, and sham surgery. Phase 2 intentions are to evaluate the safety and efficacy of three different TMJ bioengineering implants using the same rigorous method of phase 1.

## Methods

### Study Design

**Figure 1:**
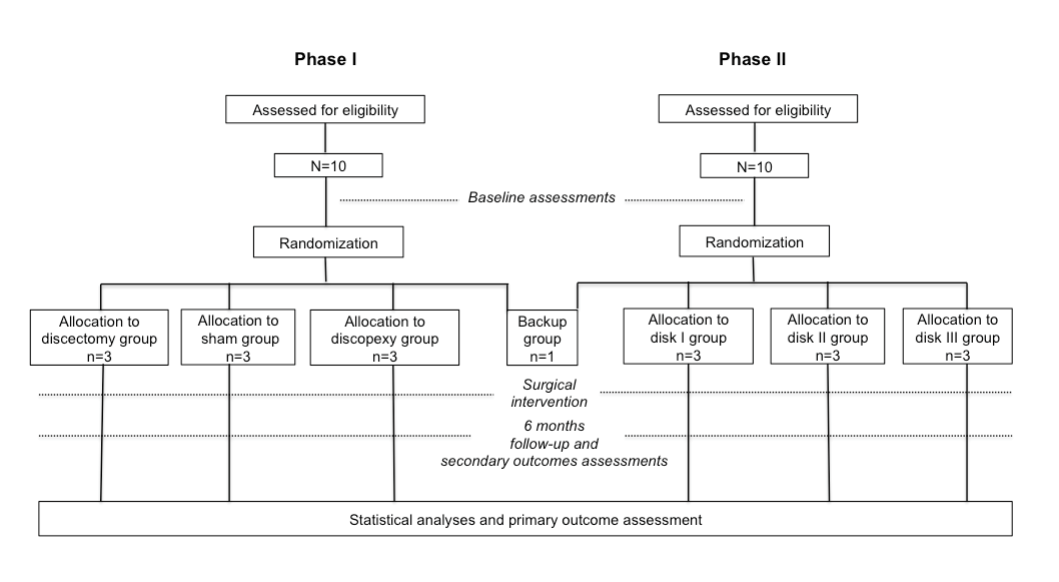
Study design.

The TEMPOJIMS is a two-phase exploratory randomized controlled preclinical trial planned to gather preliminary information to (1) evaluate a new study design for TMJ investigation; (2) evaluate the black Merino sheep animal model for TMJ investigation; (3) evaluate TMJ behavior under bilateral surgical intervention (discectomy and discopexy) using a histologic primary outcome (microscopic scoring of destructive changes in TMJ using a modified Mankin scoring system [22]), secondary imaging outcome (imaging scoring of TMJ); (4) testing the applicability of pilot secondary outcomes predominantly for ruminant kinetics; and (5) obtain a baseline for interpretation of TMJ disk bioengineering implants results. Phase II is aimed to test safety and efficacy of three different bilateral TMJ bioengineering disk implants (Figure 1). Outcome evaluators and analysts are blinded for surgical assessments.

Major institutions involved in this study are (1) Lisbon Faculty of Medicine for study design, coordination, and statistical analysis; (2) Interdisciplinary Centre of Research in Animal Health in Faculty of Veterinary Medicine for histological preparation and veterinary support of all animals; (3) Centre for Rapid and Sustainable Product Development for bioengineered disk implants (disks I and II); (4) Bioengineering, Surgery, Chemical Engineering, Mechanical Engineering and Materials Science, University of Pittsburgh, for bioengineered disk implants (disk III); (5) Department of Oral and Maxillofacial-Head and Neck Surgery, University Hospital Infanta Cristina, Badajoz, Spain, for surgical support; (6) Institute of Bone and Joint Research-Northern Sydney Local Health District-Sydney Medical School Northern, University of Sydney, Australia, for histological analysis; and (7) Radiology Department of Santa Maria Hospital, Lisbon, Portugal, for imaging analysis.

### Animal Model

A variety of strains/breeds of sheep have been used in TMJ investigations. To decrease biological variability, the authors recommended black Merino sheep as the animal model to conduct the study [20]. As recommended, the authors proposed to use “sheep skeletally mature” at ≥2 years of age [23]. The inclusion criteria are certified black Merino sheep, adult (age 2-5 years), female, and in good health condition (veterinary check-up is performed on all animals). Regarding the animal ethical considerations, the study design was approved by the Portuguese National Authority for Animal Health registered with number 026618. The study design and organization respect the Animal Research: Reporting of In Vivo Experiments (ARRIVE) guidelines.

### Baseline and Follow-Up Evaluation

**Figure 2:**
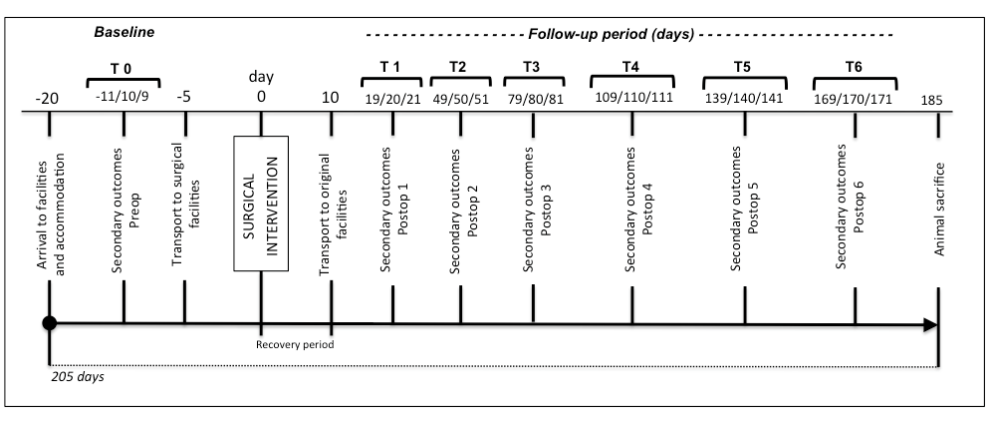
Study flowchart.

The baseline and follow-up evaluations are outlined at particular time points (Figure 2). Pilot secondary outcomes and weight are measured at days 11, 10, and 9 before surgery (details on secondary outcomes are reported in outcomes measures). Transportation to surgical facilities is performed 5 days before surgery to avoid animal stress and allow familiarization to the temporary facilities. Head computerized tomography (CT) scan is performed on the day of surgery taking advantage of preanesthesia sedation. Ten days after surgery, animals are transported to TEMPOJIMS main facilities. Days 19, 20, and 21 after surgery, the follow-up secondary outcomes start to be recorded every 30 days for 6 months (Figure 2). At the end, animals are sacrificed and a new CT scan is performed to measure the imaging outcome and to begin the histologic preparation.

### Randomization, Allocation, and Blinding

The randomization is performed by a statistical group not involved in the outcome assessments, managed by Lisbon Faculty of Medicine. Allocation to each randomized group is performed preoperatively by sealed envelope and separately for phase 1 and phase 2 of the study. The surgical team is not blinded to treatment allocation given the type of intervention; however, surgical team members are not involved in outcome assessments. All outcome evaluators are blinded to intervention. In phase 1, 10 sheep are allocated to the intervention group: sham surgery group (n=3), discectomy group (n=3), discopexy group (n=3), and backup group (n=1). The backup sheep is planned to be used if death occurs due to anesthesia or another complication not related to the surgical intervention. In phase 2, 10 sheep are randomly assigned to disk I group (n=3), disk II group (n=3), disk III group (n=3), and backup group (n=1) (Figure 1).

### Intervention Phase

#### Anesthesia Protocol

Fasting and water restriction are required 24 hours before surgery. Sedation is performed with diazepam (0.5 mg/kg iv), followed by anesthesia induction with ketamine (5 mg/kg iv). Oral intubation is performed and anesthesia is maintained with isoflurane (1.5% to 2%). To assure animal analgesia, meloxicam (0.5 mg/kg iv, bid) is administered on surgery day and during 4 days postoperatively. Antibiotic prophylaxis with amoxicillin and clavulanic acid are used for 5 days.

#### Surgical Intervention Protocol for Phases 1 and 2

##### Phase 1

Bilateral discectomy (n=3): under general anesthesia, the surgical team perform a preauricular skin incision and a blunt dissection of the soft tissue covering the joint. The joint area is disclosed and the articular capsule is incised. The disk and its attachments are identified. The medial, anterior, posterior, and lateral disk attachments are detached and discectomy is performed. The wound is closed in layers.

Bilateral discopexy (n=3): under general anesthesia, the surgical team perform a preauricular skin incision and a blunt dissection of the soft tissue covering the joint. The joint area is disclosed and the articular capsule is incised. The disk and its attachments are identified. The lateral and posterior disk attachments are detached and sutured with poly- *p*-dioxanone (PDS) 3/0. The wound is closed in layers.

Sham surgery (n=3): under general anesthesia, the surgical team will perform a preauricular skin incision and a blunt dissection of the soft tissue covering the joint. The capsule is not incised. The wound is closed in layers.

##### Phase 2

Disk I (n=3): under general anesthesia, the surgical team perform a preauricular skin incision and a blunt dissection of the soft tissue covering the joint. The joint area is disclosed and the articular capsule is incised. The disk and its attachments are identified. The medial, anterior, posterior, and lateral disk attachments are detached and discectomy is performed. The disk I is introduced into the articular space and sutured in the lateral attachments. The wound is closed in layers. Disk I will be an alternative biomaterial and for intellectual reasons cannot be revealed in this paper.

Disk II (n=3): under general anesthesia, the surgical team perform a preauricular skin incision and a blunt dissection of the soft tissue covering the joint. The joint area is disclosed and the articular capsule is incised. The disk and its attachments are identified. The medial, anterior, posterior, and lateral disk attachments are detached and discectomy is performed. The disk II is introduced into the articular space and sutured in the lateral attachments. The wound is closed in layers. Disk II will be a porous poly(glycerol sebacate) (PGS) scaffold reinforced with polycaprolactone (PCL).

Disk III (n=3): under general anesthesia, the surgical team perform a preauricular skin incision and a blunt dissection of the soft tissue covering the joint. The joint area is disclosed and the articular capsule is incised. The disk and its attachments are identified. The medial, anterior, posterior, and lateral disk attachments are detached and discectomy is performed. The disk III is introduced into the articular space and sutured in the lateral attachment. The wound is closed in layers. Disk III will be a porous PGS scaffold prepared by a modified salt fusion method. Briefly, ground salt particles (150 mg) with a size range of 25 to 32 µm will be placed into a 3-D printed mold. The mold will be transferred to an incubator at 37°C and 90% relative humidity for 1 hour. The fused templates of salt particles will dry in a vacuum oven at 90°C and 100 millitorr (mTorr) overnight, removing salt cake carefully from the mold before further processing. Fresh-made PGS dissolved in tetrahydrofuran (THF; 20 wt%, 380 µL, salt:PGS=2:1) added to the salt cake, and the THF is allowed to evaporate completely in a fume hood for 30 minutes. The salt cake is transferred to a vacuum oven and cured at 150°C and 100 mTorr for 24 hours. The resultant PGS-impregnated salt templates are soaked in deionized water for 4 hours, and then replaced with water for 4 hours, with water exchange every 4 hours during the first 12 hours. After the 12-hour water bath, scaffolds are transferred to deionized water for another 24 hours with water exchange every 8 hours. The resultant scaffolds are frozen down at –80°C and then the lyophilization process is applied.

Ten days for recovery is contemplated for wound care and postoperative medication (see Figure 2).

### Outcome Measures

**Figure 3:**
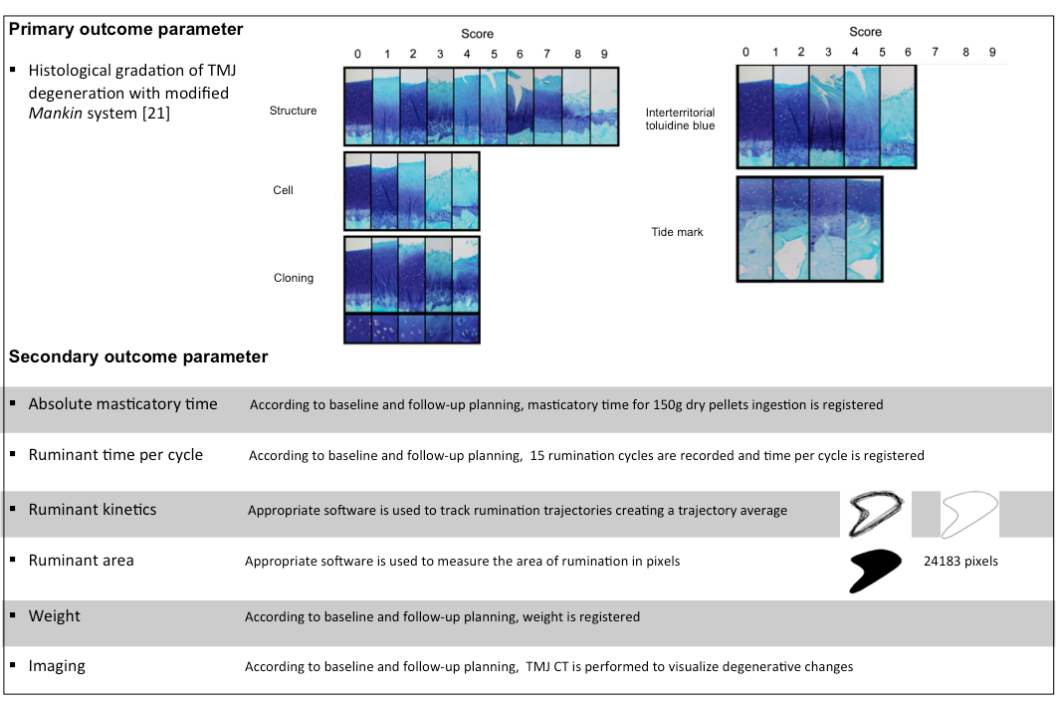
Primary and secondary outcome parameters.

The primary outcome is the microscopic scoring of destructive changes in the TMJ using a modified Mankin scoring system [22]. Secondary outcomes are imaging scoring of TMJ destructive changes, absolute masticatory time, ruminant time per cycle, ruminant kinetics, ruminant area, and sheep body weight. Primary and secondary outcome parameters are outlined in more detail in Figure 3.

#### Primary Outcome

The goal is to evaluate histologic gradation of TMJ destructive changes. The time point is 6 months following surgical intervention.

Six months after surgery, the TMJ is removed using a necropsy bone oscillatory saw according to the following anatomic references: cranial (cranial aspect of coronoid process in the union region of the zygomatic process), caudal (external to acoustic meatus), dorsal (reference is established to the squamous temporal bone), and ventral (reference is fixed 2 cm below the acoustic meatus in the zone of stylohyoid angle). The joints are fixed in 10% buffered formalin for 24 hours and stored in 70% ethanol. Decalcification is obtained by immersion in 10% formic acid in 5% formalin for up to 20 days, after which the articulations are cut sagittally through the whole condyle. After decalcifying, TMJ articulations are immersed in three graded methyl salicylate/paraffin mixtures and cut sagittally through the lateral into the central part of the TMJ. Histological sections are sent to Sydney Institute of Bone and Joint Research for histological scoring using a modified Mankin scoring system [22]. This assessment is performed and classified independent by two histologists who will be blinded to intervention. A third histologist will act as arbiter in case of disparity.

#### Secondary Outcomes

The features evaluated are imaging analysis, absolute masticatory time, ruminant time per cycle, ruminant kinematics, ruminant area, and sheep weight (see Multimedia Appendices 1 and 2). Time point is every month following surgical intervention for a total of 6 months.

**Figure 4:**
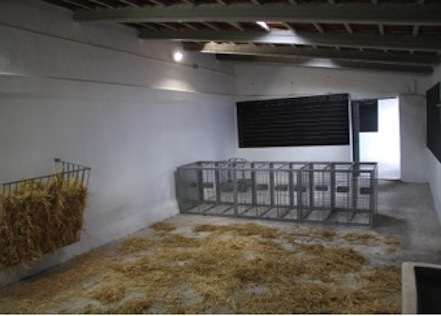
TEMPOJIMS main facilities.

To measure secondary outcomes, a specific cage (see Figure 4) was built with a frontal window and a feeder.

Imaging analysis: preoperative CT is performed on all sheep. After animal sacrifice, TMJ blocks are scanned by CT and imaging evaluation is performed using the criteria and score described in Table 1.

**Table 1:** TEMPOJIMS imaging evaluation criteria.

Items	Criteria	0 (no change)	1 (mild change)	2 (moderate change)	3 (severe change)
Shape	Change of joint form	May include reformed joint	Small changes; this change may include ≤2 osteophytes	Moderate changes; multiple osteophytes	Severe changes and outgrowth; marginal proliferation
Condyle erosion	Concavity in cortical	This stage includes normal joint with no signs of condyle erosion	Erosion in one-third of joint surface	Erosion in two-thirds of joint surface	Erosion over all joint surface
Temporal erosion	Concavity in cortical	This stage includes normal joint with no signs of temporal erosion	Erosion in one-third of joint surface	Erosion in two-thirds of joint surface	Erosion over all joint surface
Condyle sclerosis	Cortical thickening of condyle	This stage includes normal joint with no signs of condyle sclerosis	Sclerosis in one-third of joint surface	Sclerosis in two-thirds of joint surface	Sclerosis over all joint surface
Temporal sclerosis	Cortical thickening of temporal fossa	This stage includes normal joint with no signs of temporal sclerosis	Sclerosis in one-third of joint surface	Sclerosis in two-thirds of joint surface	Sclerosis over all joint surface
Condyle marrow	Change of underlying trabecular bone	This stage includes normal joint with no change of condyle trabecular bone	Sclerosis in less than half of trabecular bone	Sclerosis in half of trabecular bone	Sclerosis in all trabecular bone
Temporal marrow	Change of underlying trabecular bone	This stage includes normal joint with no change of temporal trabecular bone	Sclerosis in less than half of trabecular bone	Sclerosis in half of trabecular bone	Sclerosis in all trabecular bone
Calcification	Development of calcification across joint space	No calcification across joint space	Calcification in one-third of joint surface	Calcification in two-thirds of joint surface	Bony fusion across joint space
Global appreciation		Normal joint	In general, mild changes	In general, moderate changes	In general, severe changes

This assessment is performed and classified independently by two experienced radiologists who will be blinded to intervention. A third radiologist will act as arbiter in case of disparity.

Absolute masticatory time: respecting the flowchart (Figure 2), at 9:00 am the animals are placed in individual cages. A dose of 150 grams of dry pellets (Rico Gado A3) are introduced in the feeder and the time until they eat all the pellets is measured with a chronometer (see Multimedia Appendix 1).

Ruminant time per cycle: respecting the timetable (Figure 2), we record 15 ruminatory cycles approximately 4 hours after 150 gram feeding. We use a Canon 7D video camera and images with 25 frames per second. Then, the number of frames per cycle are divided by 25 to obtain time in seconds per cycle (see Multimedia Appendix 2).

Ruminant kinetics: we use the software Foundry Nuke (2D tracking) to perform the ruminatory tracking and to obtain the ruminatory cycle average. With the software After Effects *,* we convert the 2-D tracking into a geometric form (see Multimedia Appendix 2).

Ruminant area: we determine the average of 15 cycles and create a geometric form. Using the software Image J *,* we perform a quantitative measure in pixels of the ruminant area average.

Weight: according to the timetable, after eating 150 grams of dry pellets the sheep are weighed (see Multimedia Appendix 1).

All assessments are performed by researchers who are blinded to surgical intervention.

### Statistical Analyses

All statistical analyses will be performed using the SPSS version 22 (IBM Corp, Armonk, NY, USA). A cross-sectional analysis will be performed to compare the outcome variables in the three levels of the independent variable before and after the randomized treatment group assignment. In the cross-sectional analyses, one-way analysis of variance (ANOVA) will be performed, after testing all the assumptions. For longitudinal analysis, one-way ANOVA with repeated measures will be performed taking as within-subjects effects observations after surgery (months 1 to 6). Fisher least significant difference will be performed as post hoc tests to check for significant differences for the different treatments.

### Reporting of Adverse Events

Adverse events related to the study will be considered, including (1) anesthesia events: idiopathic death, pneumothorax, other complications related to anesthesia; (2) surgical technique: massive bleeding, condylar fracture, other complications related to surgical technique; and (3) postoperative events: TMJ infection, suture dehiscence, decreased appetite, facial paresis, decreased rumination, decreased weight.

## Discussion

This study investigates the effects and adverse effects of (1) bilateral discectomy, (2) bilateral discopexy, and (3) bioengineered disk implants. Although this preclinical study will primarily serve as a pilot study, we expect to gain a better understanding of the morphologic and histologic changes in TMJ and implications in masticatory kinetics.

So far, results on discectomy are conflicting. Previous preclinical studies in this field [24-33] have used the contralateral unoperated side as a control and different animal models ranging from mice to a canine model. Using the contralateral side as a control can be inappropriate considering contralateral overload influence. Theoretically, we expect to reduce this bias using a bilateral approach. Animal variability in the different studies is a warning about the importance of using the same animal model in further studies regarding TMJ implant investigations. Therefore, our group performed a previous study considering black Merino sheep as a promisor animal model for studies regarding TMJ disk implants investigation, TMJ prosthesis, and TMJ osteoarthritis model. To increase the quality of TEMPOJIMS the authors will use a sham surgery control group.

We expect to obtain valuable information related to the phase 1 discopexy group regarding if the surgical approach promotes intra-articular damage. This can improve future conclusions about attributing possible damage to the intervention itself instead of the TMJ implant. This question is important considering that a surgical approach to place TMJ implants in phase 2 will be required. Again, using a bilateral intervention could reduce a possible bias.

Most preclinical studies have focused on gross morphological/histological assessments and were not designed to characterize the fundamental altered joint movement (kinetics) or functional consequences. In this study, we include pilot secondary outcomes to evaluate changes in ruminant kinetics. We expect to correlate the primary with the secondary outcomes to understand if they can be used in future TMJ studies. It may be interesting to understand several items:

1. Are there differences regarding masticatory time in the disk groups versus discectomy and discopexy?

2. Is there a correlation between histologic and imaging and kinetics results?

3. Does the ruminant area and geometry change when performing different interventions?

4. Is there a difference regarding ruminant kinetics in the disk groups versus discectomy and discopexy?

5. Do TMJ implants accelerate osteoarthritis?

Concerning phase 2, the choice of biomaterial is critical. The TMJ implant will be exposed in a mechanical, stressful environment with a limited blood supply that can limit cell migration and in situ regeneration. Testing three different bioengineering discs in vivo and correlating in vitro with in vivo behavior can seriously improve bioengineering strategies to achieve a safe and efficacious TMJ disk implant for humans.

The main strength of this study is the animal model proposed; the conventional and pilot outcomes described; the study design with a randomized, blinded, and placebo control group; and the use of bilateral surgical procedures. Potential limitations of the study include the relatively small sample size. If this study confirms the feasibility of the proposed protocol and initial efficacy of the TMJ disk implants planned, a larger preclinical trial would be warranted to further determine the effectiveness of these discs and promote translation of animal evidence to clinical practice in humans.

### Trial Status

At the time of submission, the surgical interventions of phase 1 were ongoing at Faculdade de Medicina Veterinária de Lisboa and TEMPOJIMS facilities in Portugal.

## Appendix

Multimedia Appendix 1 Outcomes assessments in TEMPOJIMS main facilities, absolute masticatory time and weight.

Multimedia Appendix 2 Outcomes assessments in TEMPOJIMS main facilities. After recording 15 ruminant cycles with a Canon 7D Video Camera we used the software Foundry Nuke (2D tracking) to make the ruminant tracking to obtain the ruminant cycle average in each time period.

## Abbreviations

CT: computerized tomography
PCL: polycaprolactone
PDS: poly-p-dioxanone
PGS: poly(glycerol sebacate)
TEMPOJIMS: Temporomandibular Joint Interposal Material Study
THF: tetrahydrofuran
TMD: temporomandibular joint disorders
TMJ: temporomandibular joint
